# Prediction of malaria using deep learning models: A case study on city clusters in the state of Amazonas, Brazil, from 2003 to 2018

**DOI:** 10.1590/0037-8682-0420-2021

**Published:** 2022-08-05

**Authors:** Matheus Félix Xavier Barboza, Kayo Henrique de Carvalho Monteiro, Iago Richard Rodrigues, Guto Leoni Santos, Wuelton Marcelo Monteiro, Elder Augusto Guimaraes Figueira, Vanderson de Souza Sampaio, Theo Lynn, Patricia Takako Endo

**Affiliations:** 1 Universidade de Pernambuco, Programa de Pós-Graduação em Engenharia da Computação, Recife, PE, Brasil.; 2 Universidade Federal de Pernambuco, Centro de Informática, Recife, PE, Brasil.; 3 Universidade do Estado do Amazonas, Manaus, AM, Brasil.; 4 Fundação de Medicina Tropical Doutor Heitor Vieira Dourado, Manaus, AM, Brasil.; 5 Fundação de Vigilância em Saúde Rosemary Costa Pinto, Manaus, AM, Brasil.; 6 Instituto Oswaldo Cruz, Programa de Pós-graduação Stricto Sensu em Medicina Tropical, Rio de Janeiro, RJ, Brasil.; 7 Dublin City University, Dublin, Ireland.; 8 Instituto Todos pela Saúde, São Paulo, SP, Brasil.

**Keywords:** Malaria, Machine learning, Deep learning, Prediction, LSTM, GRU

## Abstract

**Background:**

Malaria is curable. Nonetheless, over 229 million cases of malaria were recorded in 2019, along with 409,000 deaths. Although over 42 million Brazilians are at risk of contracting malaria, 99% percent of all malaria cases in Brazil are located in or around the Amazon rainforest. Despite declining cases and deaths, malaria remains a major public health issue in Brazil. Accurate spatiotemporal prediction of malaria propagation may enable improved resource allocation to support efforts to eradicate the disease.

**Methods:**

In response to calls for novel research on malaria elimination strategies that suit local conditions, in this study, we propose machine learning (ML) and deep learning (DL) models to predict the probability of malaria cases in the state of Amazonas. Using a dataset of approximately 6 million records (January 2003 to December 2018), we applied *k*-means clustering to group cities based on their similarity of malaria incidence. We evaluated random forest, long-short term memory (LSTM) and dated recurrent unit (GRU) models and compared their performance.

**Results:**

The LSTM architecture achieved better performance in clusters with less variability in the number of cases, whereas the GRU presents better results in clusters with high variability. Although Diebold-Mariano testing suggested that both the LSTM and GRU performed comparably, GRU can be trained significantly faster, which could prove advantageous in practice.

**Conclusions:**

All models showed satisfactory accuracy and strong performance in predicting new cases of malaria, and each could serve as a supplemental tool to support regional policies and strategies.

## INTRODUCTION

Malaria is a curable, life-threatening disease caused by parasites. It is transmitted to people through the bites of infected female *Anopheles* mosquitoes. For non-immune individuals, symptoms usually appear 10-15 days after the infective mosquito bite and can progress to severe illness if left untreated^1^ ^,^ ^2^ ^,^ ^3^. The World Health Organization (WHO) recently estimated that 229 million cases of malaria and 409,000 deaths occurred in 2019^4^. Malaria poses significant social and economic burdens; estimates suggest that over 52 million disability-adjusted life years have been lost due to malaria worldwide^5^. Research suggests that the reduction of the malaria burden is associated with increased household spending^6^ and household consumption^7^, higher incomes for adults^8^, increased GDP^9^ ^,^ ^10^, greater wealth accumulation^11^, less work disability, and new forms of occupation^12^ ^,^ ^13^ as well as improved health, well-being, and quality of life.

Conditions suitable for the propagation of malaria exist in many regions worldwide. For example, over 138 million people are at risk of contracting malaria in Central and South America^14^. Although the number of cases and deaths in Brazil are in decline, in 2018, approximately 42 million people were at risk of malaria, and 232,000 cases were recorded^15^. Epidemiological studies suggest three discrete malaria transmission systems seem to function in Brazil, related respectively to the Amazon rainforest, the Atlantic rainforest, and the Brazilian coast. However, 99% of all malaria cases are located in the Amazon rainforest^16^. In 2015-2016, the states of Amazonas and Acre together reported 60-70% of malaria cases in the Amazonian region and Brazil as a whole^16^.

The persistent high rates of malaria in these regions have been variously attributed to several different factors, including anthropogenic environmental changes, human migration (including internal population movements and migration from other countries), and living standards^16^. Despite the high number of cases, the number of deaths is low, less than 30^15^. This is largely due to successive malaria control intervention programs such as the Amazon Basin Malaria Control Programme, the National Malaria Prevention and Control Programme, and the Plan for Elimination of Malaria in Brazil. Notwithstanding the progress in reducing the number of cases of malaria and malaria-related deaths, both the direct and indirect impact of malaria infection in the Amazon region remains significant^17^. Despite significant efforts and achievements in the control of malaria, further progress may be retarded due to threats of drug and insecticide resistance, the instability of international funding for malaria control, imported malaria from other countries, expansion of economic frontiers, and the falling cost-effectiveness of traditional interventions^13^. Indeed, recent work suggests that new scientific interventions to reduce mosquito biting and better insecticides should be complemented by research on the practical implementation of these methods to adapt strategies to suit local conditions^14^ ^,^ ^18^. Both demographic and epidemiological analyses of data suggest substantial heterogeneity and spatial clustering in the Amazon basin^18^ ^,^ ^19^. Consequently, there have been calls for intervention strategies targeting specific regions, potentially at lower administrative levels, or risk groups^19^. 

This situation is complicated by the COVID-19 pandemic, which introduces additional competition for funding for malaria control interventions and leads to challenging social and economic conditions^20^ ^,^ ^21^. In such situations, accurate data on future human resource requirements for spraying and treatment based on likely malaria cases by region is critical for managing disease control and mitigation where resource availability may be constrained due to social distancing, self-isolation, worker safety, or funding.

Statistical methods and machine learning (ML) models have been proposed to identify the distribution of malaria cases and vectors in India^22^ ^,^ ^23^ ^,^ ^24^, China^25^, and Thailand^26^ ^,^ ^27^. These studies use classical statistical methods and their capacity for generalization to other contexts is limited owing to the malaria vectors examined and geographic idiosyncrasies. In contrast, few studies have considered the use of deep learning (DL) to predict the distribution of malaria vectors and cases, especially for the Amazon region specifically.

Some ML models have been proposed to identify the distribution of malaria cases and vectors, particularly in Asia. Moyes et al.^22^ used data analysis and a boosted regression tree model to identify the distribution of host monkeys and mosquito vectors of the parasite *P. knowlesi*. This parasite is the leading cause of malaria in Malaysia. The authors analyzed the relationship between these species and potential environmental variables such as forest cover. Their findings suggest that the relative probability of host macaque species and members of the *Leucosphyrus Complex* occurring in disturbed forest areas such as plantations timber concessions, and vegetation mosaics brings species into close contact with human activities. This has implications for both mitigation and eradication plans in addition to treatment and economic development.

Sarkar et al.^23^ proposed the use of time-series models based on epidemiological data. They used autoregressive integrated moving average (ARIMA), generalized autoregressive conditional heteroskedastic (GARCH), and random walk models to predict the incidence of malaria caused by the parasite *P. vivax* in Chennai, India. Their results suggested that the models chosen fit well with epidemiological data and provided useful predictions for malaria incidence, where these models have not been used extensively with appropriate parameter choices. This work could provide inputs for the design of malaria control programs.

In general, most methods reported in the relevant literature have adopted classical regression models and conventional ML techniques to predict the incidence of malaria. Chae et al.,^28^ proposed a DL model along with other methods to forecast three different infectious diseases in South Korea, including malaria, chickenpox, and scarlet fever. Four types of data (search query data, social media big data, temperature, and humidity) were used to predict cases, and their proposed deep learning models outperformed the traditional ARIMA^28^.

It is important to note that while *P. vivax* is widespread in Brazil, *P. falciparum still* plays an important role in malaria transmission, and the studies above may not be generalizable to Brazil due to the difference in malaria vectors and environmental context. While a limited number of studies have been conducted that focus on Brazil, they typically focus on mapping the geospatial patterns of malaria using a variety of techniques, including pattern detection using normalized difference vegetation index^29^, Poisson normal models^30^, free-form covariance models^31^, and Bayesian and Markov chain Monte Carlo methods^32^, along with several others. Cunha et al.^33^ focused on the municipality of Cantá in the state of Roraima, Brazil to measure the risk of malaria cases according to the annual parasitic index (IPA). Cantá has one of the highest index values in the country. The authors proposed a multilayer artificial neural network (feedforward) using a database with records from 2003 to 2008.

In this work, we consider the following research question. "Does grouping cities by confirmed cases of malaria improve the performance of ML/DL methods for predicting malaria cases in the state of Amazonas?” This study includes two main contributions. First, we evaluated DL models to predict the occurrence of malaria in the state of Amazonas, Brazil. The present work is among the first DL studies on malaria in Brazil, and we utilized a substantial clinical dataset. DL models may contribute to better prediction results and consequently lead to the development of more effective intervention strategies. The second contribution of this work relates to the use of clustering models to group cities based on similarity of malaria incidence.

## METHODS

### Data set

The state of Amazonas is the largest in Brazil, with an area of over 1,559,161 square kilometers^34^, and is one of the largest country subdivisions in the world, comprising 62 cities. It is dominated by tropical jungle, having the largest area of preserved forest among the states in the region.

**FIGURE 1:**
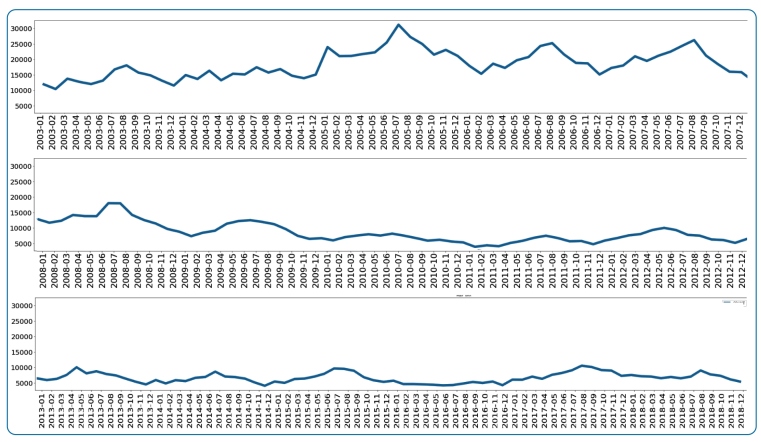
Monthly time series of malaria cases for the State of Amazonas from 2003 to 2018.

We used data from the *Sistema de Informação de Vigilância Epidemiológica de Malária* (SIVEP-MALARIA), which is a specific information system for reporting malaria cases in the Brazilian Amazon. The dataset includes data related to malaria cases that occurred from January 2003 to December 2018 in the state of Amazonas, comprising approximately 6 million records. Figure 1 presents the time series for the number of malaria cases per month in the state of Amazonas. Between 2003 and 2007, the number of cases was higher than in the preceding years, reaching 30,000 cases in July 2005. After 2008, the number of cases decreased.

We used the holdout validation method^35^ to carry out the experiments with city clusters. We selected 80% of the available historical data (from January 2003 to October 2015) to train the model, and 20% (October 2015 to December 2018) to perform testing. We conducted experiments for each technique ten times to ensure the statistical validity of the results. We then calculated the average root mean square error (RMSE) and standard deviation for each model. 

### Clustering

Clustering techniques divides the samples of a dataset into groups according to the similarity of the characteristics of each element^36^. The *k*-means algorithm is among the most well-known data clustering methods. It partitions a predefined number of clusters *k* using an unsupervised classification. The algorithm compares elements based on the Euclidean distance between average values of the data^37^.

**FIGURE 2:**
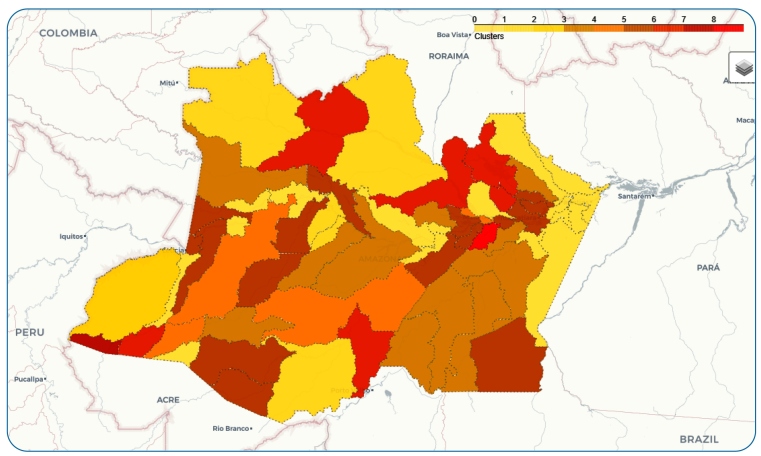
Clusters resulting from the *k*-means algorithm (*k* = 9). Each color represents a cluster as described in Table 1. Monthly time series of malaria cases for the State of Amazonas from 2003 to 2018.

In our study, the clusters were created using the k-means algorithm, considering the mean, median, and maximum cases of malaria per 1,000 inhabitants as statistical features^38^. For convenience, we defined nine clusters (k=9) based on the health regions in the State of Amazonas, as shown in Figure 2. Cities in Amazonas are marked in colors based on the cluster to which they belong. As the clustering was performed according to statistical data of reported malaria cases, cities in a given cluster need not necessarily be geographically close to each other.

**TABLE 1:** RMSE results by cluster.

	LSTM	GRU	Random Forest
**Cluster 1 (n = 17)**	0.0384 (±0*.*0099)	**0.0363 (±0*.*0005)**	0.0368 (±0*.*0001)
**Cluster 2 (n = 4)**	0.0565 (±0*.*0106)	0.0499 (±0*.*0022)	0.0588 (±0*.*0004)
**Cluster 3 (n = 1)**	0.0543 (±0*.*0125)	0.0483 (±0*.*0021)	0.0556 (±0*.*0001)
**Cluster 4 (n = 11)**	0.0406 (±0*.*0114)	0.0383 (±0*.*0007)	0.0398 (±0*.*0002)
**Cluster 5 (n = 5)**	0.1707 (±0*.*0560)	0.1564 (±0*.*0017)	0.1543 (±0*.*0004)
**Cluster 6 (n = 16)**	0.0477 (±0*.*0085)	0.0450 (±0*.*0015)	0.0492 (±0*.*0002)
**Cluster 7 (n = 6)**	0.0863 (±0*.*0239)	0.0782 (±0*.*0008)	0.0838 (±0*.*0003)
**Cluster 8 (n = 1)**	0.0622 (±0*.*0131)	0.0586 (±0*.*0005)	0.0618 (±0*.*0003)
**Cluster 9 (n = 1)**	0.0127 (±0*.*0011)	0.0131 (±0*.*0008)	0.0134 (±0*.*0000)

RMSE results for each city cluster in the State of Amazonas. RMSE results by cluster (average of 10 repetitions). **±:** Indicates notation for standard deviation.

### Metrics

To quantitatively assess the ML models, as per^24^, we used the root mean square error (RMSE) owing to its advantages in terms of unbiased errors compared to other metrics^39^ such as the mean absolute error (MAE) model. The RMSE can be defined as:

$$RMSE=\sqrt{\frac{1}{N}\sum_{t=1}^{N}\;{\left(y_{t}-{\overset{ˆ}{y}}_{t}\right)}^{2},}$$

where *y*_*t*_ is the actual value, *ŷ*_*t*_ is the value predicted by the model, and *N* is the value given the number of measured points or days (4,667 points for the training dataset and 1,169 points for the test dataset)^40^. The smaller the RMSE, the better the predictions of the model.

Tests were repeated ten times with long short-term memory (LSTM), gated recurrent unit (GRU), and random forest models, and then the RMSE arithmetic mean and standard deviation were calculated based on the time-series data (number of cases of malaria) normalized between 0 and 1. The main objective of the data normalization method was to produce better quality data to feed the learning algorithms. Time-series data can take on a wide range of values, so such datasets need to be scaled to the same range of values to improve the learning process^41^.

To create the prediction models, we considered three different approaches, including LSTM and GRU as DL techniques, and random forest as a conventional ML technique.

### LSTM and GRU models

Recurrent neural networks (RNNs) are a variation of traditional neural networks that are capable of working with previous connections, thus allowing decision-making based on both preceding and recent information. LSTM and GRU are special types of RNN that specifically address the gradient dissipation problem. This dissipation is a failure that occurs for excessively long data sequences, which results in an increase in gradient values along the sequence’s growth.

The architectures of both LSTM and GRU are very similar. LSTM and GRU networks both include internal mechanisms called gates, designed to control the flow of information^42^. These gates can identify which data is important to retain during the learning process and which data can be discarded. This process helps to maintain important information during a longer chain of data compared to traditional RNNs^43^.

We consider two DL models to predict the occurrence of malaria - an LSTM and GRU. Both models have the same architecture, composed of two layers (LSTM or GRU), both with fifty units per layer. Each LSTM or GRU layer is followed by a dropout layer, with parameters set to 20% chance of readjusting weights to reduce overfitting followed by a layer fully connected with a unit that provides the malaria forecast as an output. The parameters (such as the number of layers and units) were chosen empirically. After each recurring layer (LSTM and GRU), we use the dropout technique with a probability of 20%^44^.

### Random Forest model

The random forest algorithm involves the construction of specialized decision trees^45^. It can be applied to various prediction problems, having few parameters to adjust. The method is simple to use, and is known for its accuracy and ability to deal with small sample sizes^46^. It has been widely used in the context of malaria, including object detection in malaria images^47^ ^,^ ^48^, quantification of malaria parasitemia in microscopy^49^, and reactive case detection^50^, among other applications. It has also been used as a comparator in malaria case detection and classification studies using different techniques^28^ ^,^ ^51^ ^,^ ^52^.

Random forest model can be defined in a simple manner by two parameters: the number of decision trees and their maximum depth. The number of decision trees used in this work was equal to 100, and this value was based on repeated tests conducted to verify the best performance according to this parameter. The maximum depth was selected for its default value (zero) to expand the nodes until all leaves contained as few samples as possible.

## RESULTS

Table 1 presents the average of the RMSE results for city clusters in the state of Amazonas, the number of municipalities contained, and their respective standard deviations. The GRU model exhibited the best RMSE for the majority of city clusters (7 of 9), varying from 0.0131 (Cluster 9) to 0.0782 (Cluster 7). The exceptions were Cluster 5, on which the random forest model obtained the best RMSE (0.1543), and Cluster 9, on which the LSTM presented the best RMSE (0.0127). In general, the best RMSE results were achieved for Cluster 9, and the worst for Cluster 5.

To confirm the results obtained, we performed a statistical test to compare the results of the proposed models. We use the Diebold-Mariano (DM) test, a two-sample hypothesis test, to compare the prediction of two predicted time series. By definition, the DM test gives negative results when the predicted time series on the left achieves a better result and provides a positive value when the predicted time series on the right achieves a better result^53^.

**FIGURE 3:**
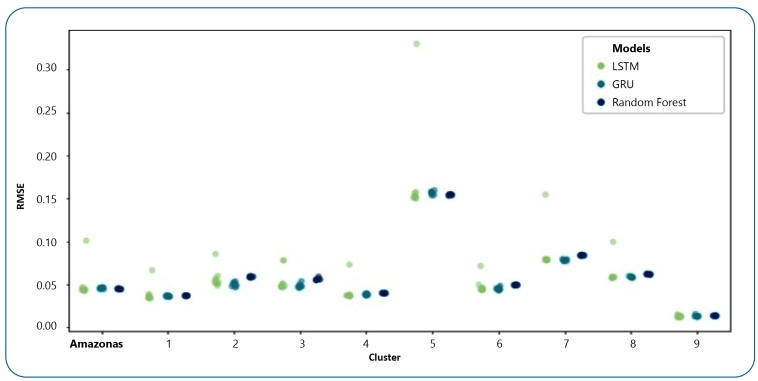
Scatter plot for the three models used in tests by city cluster (average of 10 repetitions).

Based on the results presented in Table 1, the DL models outperformed the random forest model in all clusters with the exception of Cluster 5. Cluster 5 exhibited the greatest variation in the number of malaria cases in the time series, no clear pattern was evident (Figure 3). Such behavior impacts the performance of DL models, which rely on learning patterns in the data to make predictions. This reinforces the earlier conclusions from the cluster analysis in that the LSTM model may be considered more suitable for predicting cases of malaria using data with few oscillations, whereas the GRU model performs better at predicting cases where there is greater variability.

**FIGURE 4:**
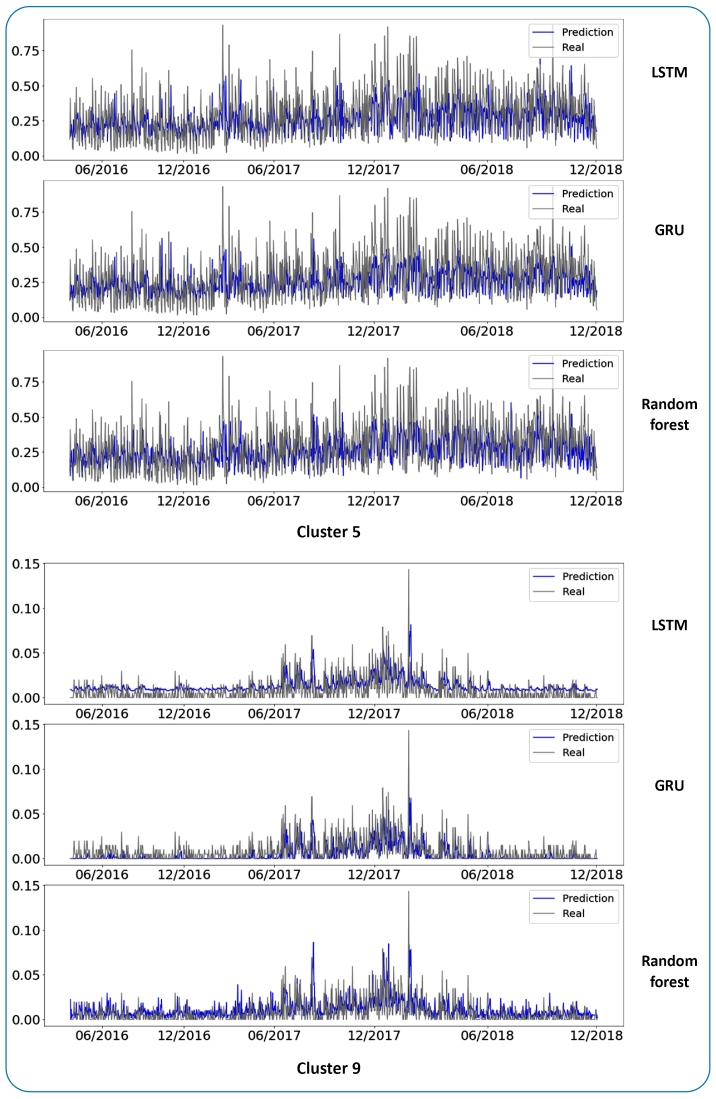
Prediction results by city cluster.

The LSTM model exhibited a greater standard deviation than the other models. Figure 4 presents the prediction results by city cluster for each model. Figure 3 presents the dispersion graphs for the RMSE results for each city cluster. The LSTM model showed the highest dispersion. However, the results were very similar. Consequently, the Diebold-Mariano test was also conducted for these results.

Table 2 presents the DM test results by city cluster. The results suggest that the LSTM and GRU models outperformed the random forest model in most of the clusters. Compares the LSTM and random forest models, the former outperformed the latter in seven of the nine clusters, and the GRU model outperformed the random forest model in eight of the nine clusters.

**TABLE 2:** Diebold-Mariano results by cluster.

Cluster	LSTM vs. GRU	LSTM vs. RF	GRU vs. RF
**Cluster 1 (n = 17)**	-6,28	-4,57	-1,45
**Cluster 2 (n = 4)**	4,39	9,40	-8,05
**Cluster 3 (n = 1)**	-6,50	-10,2	-8,09
**Cluster 4 (n = 11)**	-4,87	-4,93	-1,99
**Cluster 5 (n = 5)**	2,50	2,63	0,95
**Cluster 6 (n = 16)**	-7,21	-8,96	-5,67
**Cluster 7 (n = 6)**	0,47	-4,25	-5,25
**Cluster 8 (n = 1)**	-2,57	-4,46	-3,59
**Cluster 9 (n = 1)**	-3,61	-4,19	-2,96

Diebold-Mariano test results by city cluster. Diebold-Mariano results by cluster (average of 10 repetitions).

## DISCUSSION

In this study, we analyzed the prediction of malaria cases between 2003 and 2018 by city clusters, and constructed models that exhibited improved performance, with RMSE results ranging from 0.0131 to 0.0782. The standard deviation was practically insignificant, varying from ±0*.*0001 to ±0*.*0560. From a deep learning perspective, our results are consistent with those of previous works^54^. The DM results also suggest that although the LSTM model achieved a higher RMSE based on cluster samples, the number of forecast points contributing to this error was not as high as that observed in the random forest or the GRU model. Notwithstanding the comparable performance of the LSTM and GRU methods, the latter has significantly faster training times^18^ ^,^ ^55^, which may prove advantageous in practice.

Our results also showed that the LSTM model exhibited better performance in clusters with less variability in the number of malaria cases, whereas the GRU model exhibited better results in clusters with high variability. From an epidemiological perspective, high variability can represent a more complex scenario because epidemic episodes are present (represented by peaks), reinforcing the practical applicability of our proposed GRU model. An accurate computational model to predict this variability can be a useful public health tool because policymakers can consider decisions in advance, optimizing resource allocation and planning social actions to reduce the impacts of a possible outbreak of malaria.

Based on the results, the proposed models present a highly accurate prediction of malaria cases and could serve as a supplemental tool to support regional policies and strategies^56^, considering both regional characteristics and the relevant epidemiological profile.

## CONCLUSION

Recent research has suggested any efforts to eliminate malaria depends on the incidence and effectiveness of interventions in the Amazon region due to unequal distribution of malaria incidence in Brazil,^18^. In response to calls for novel work on the adaptation of malaria mitigation and eradication strategies to suit local conditions, in this study, we have proposed ML and DL models to predict the probability of malaria cases in the state of Amazonas. Using a dataset of approximately six million records, we have evaluated random forest, LSTM, and GRU models. Our findings suggest that all models showed satisfactory accuracy and strong potential to predict new cases in city clusters. While Diebold-Mariano testing suggested that both the LSTM and GRU models achieved comparable results, GRUs have significantly faster training times, which could prove advantageous in practice.

The rapid and accurate prediction of the distribution of new cases at lower spatial resolutions, in this case by city, is an important first step in using big data analytics to estimate human disease risk and inform disease control planning at both national and lower administrative levels. Malaria in the state of Amazonas is significantly impacted by the unique socio-environmental factors associated with the Amazon rainforest. It is particularly at risk from future frontier expansion and population mobility within Brazil and from other countries. Lana et al.^18^ suggested that spatiotemporal heterogeneity in Brazilian malaria transmission requires a radical rethinking of malaria surveillance and elimination strategies in Brazil with a shift to from a ‘one-size fits all’ approach to targeted and dynamic surveillance. Our research suggests that ML and DL models can be potentially low-cost decision support tool for supporting national, regional, and local malaria control strategies.

This work involves some limitations. The source database contained data only on patients diagnosed with malaria in the state of Amazonas between 2003 and 2018. Future work can replicate and extend our work to other states in Brazil, as well as other countries where malaria is prevalent. The main objective of this study was not to locate individuals at a higher risk of malaria, but to compare computational models capable of predicting malaria cases. For this research, the number of clusters was defined by the number of health regions in the state of Amazonas (nine in total) to serve as a baseline. We only considered tests on *k*-means clusters. Consequently, the *k* value of the clusters was not evaluated for values other than nine. Future work might consider other spatially significant clustering strategies at lower spatial resolutions as well as other subpopulations.

The next stage of this research is to extend the current work to an index of risk and then consider how the sophistication of the model can be developed to consider other risk factors. In addition to the pluviometric regimes and associated seasonal changes, we plan to explore geospatial, environmental, and socioeconomic factors (including occupation), the distribution of disease vectors of varying types, and the impact of other disease control programs, such as COVID-19, on malaria control and resource management.
